# Challenges and adaptations in training during pandemic COVID-19: observations by an orthopedic resident in Singapore

**DOI:** 10.1080/17453674.2020.1786641

**Published:** 2020-07-03

**Authors:** Wei-Sheng Foong, H L Terry Teo, D H Bryan Wang, S Y James Loh

**Affiliations:** Department of Orthopaedic Surgery, Changi General Hospital, Singapore

Orthopedic surgery training in Singapore is provided by 3 Accreditation Council for Graduate Medical Education International (ACGME-I) accredited residency programs. Mandatory clinical rotations in general surgery, plastic surgery, and intensive care in a foundation year (R-1) and followed by subspecialties within a 5-year program are requirements for graduation.

In January 2020, China confirmed its first case of COVID-19 coronavirus case in Wuhan (Chen et al. 2020). This occurred 2 months before the World Health Organization (WHO) announced the disease as a global health crisis. The pandemic has affected the healthcare industry in Singapore in a myriad of ways. There was an exponential increase in the usage of personal protection devices such as masks and gloves. Measures were taken to triage surgeries to control bed usage, such as high-dependency beds, as well as to stockpile crucial anesthetic drugs in the event of a tsunami of patients who needed intensive care.

The impact of this fast-evolving and widespread pandemic has since affected all continents with the exception of Antarctica. The various control measures (ECDC 2020) implemented have changed the routines of healthcare provision. This has inadvertently affected residency training in orthopedic surgery. Several programs (Amparore et al. 2020, Nassar et al. 2020) worldwide have undertaken measures to ensure the continuity of residency training with minimal disruption. Similarly, my (“my/I” refers from now on to author W-S F) training program has made innovative changes (Schwartz et al. 2020) and adaptations with utmost emphasis on training with all the necessary safety precautions.

The following account is based on the views and observations of an individual and does not reflect that of any specific training program or organization.

## ACGME-I Orthopedic Surgery Residency Program: components and competencies

ACGME-I is guided by 3 main administrative components inclusive of clinical experience, didactic teaching, and duty hours. 6 core competencies have been identified to guide the design of a well-balanced surgical training program. These competencies include patient care, knowledge, systems-based practice, professionalism, practice-based learning, and communication skills (ACGME 2020a, 2020b).

## The 3 core components

### Clinical experience

COVID-19 spreads predominantly via aerosol droplets and contaminated surfaces (Viswanath and Monga 2020). This dictates that the healthcare provider should use various personal protection devices such as mask and gloves, coupled with strict hand hygiene. The mitigation measures (Sahu et al. 2020) also advised reduction of doctor and patient contact. A direct consequence of this measure is the postponement of non-essential surgeries and clinic appointments, resulting in a decline in clinical exposure in these areas. In spite of these measures, essential orthopedic surgeries such as fractures and spine conditions with neurological compromise continued to proceed.

### Surgery

**Figure 1.**
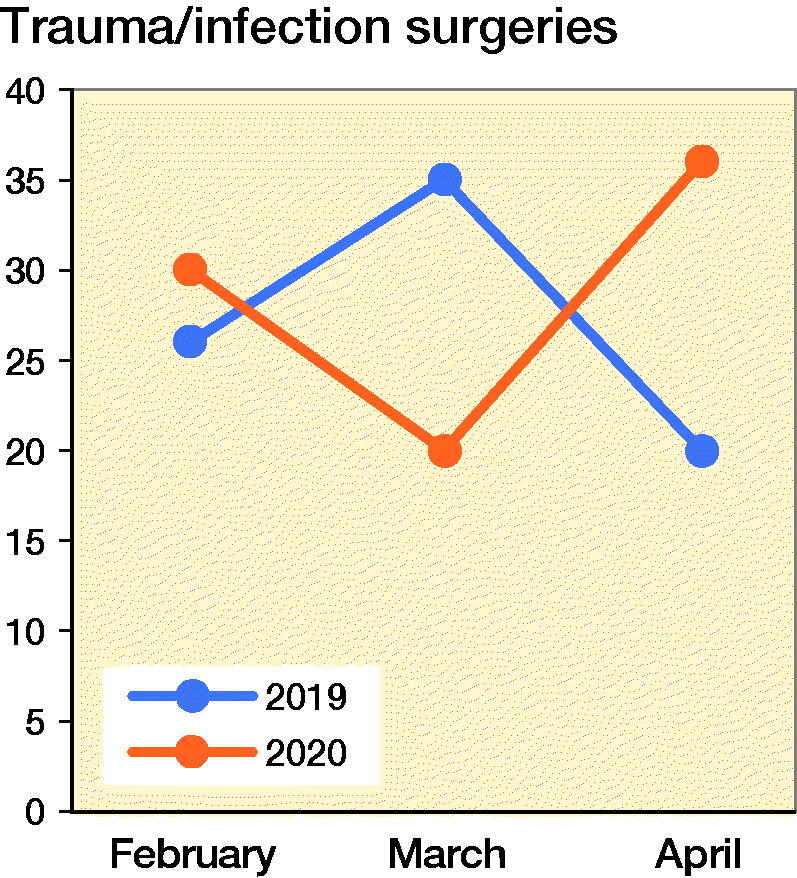
Number of trauma/infection surgeries from February to April, 2019 and 2020.

The orthopedic resident is expected to participate in 200 (ACGME 2020a) surgeries in a year. Over a period of 3 months, from February to April 2020, there was a steady and significant decline in the number of elective arthroscopic and arthroplasty surgeries. The decline in the number of listed surgeries is possibly the result of a lower demand in non-emergency conditions and advisories to postpone non-essential elective surgeries. This decline correlates inversely with the sharp climb in the number of positive COVID-19 cases reported over the same period. Figure 1 details the difference in total number of cases for the same period of 3 months from February through April in the years 2019 and 2020. It compares my experience during this period with a fellow resident under the same supervising attending consultant in 2019. There was an overall drop of 9% in the total number of surgeries.

The surgeries were categorized into tiers 1, 2. and 3. These are vetted by the Operating Theatre Management Unit (OTMU). Tier 1 is for life-threatening conditions such as tumor surgery. Fractures and musculoskeletal infections are in tier 2. Elective orthopedic surgeries such as knee replacement and knee arthroscopy are categorized as tier 3.

**Figure 2.**
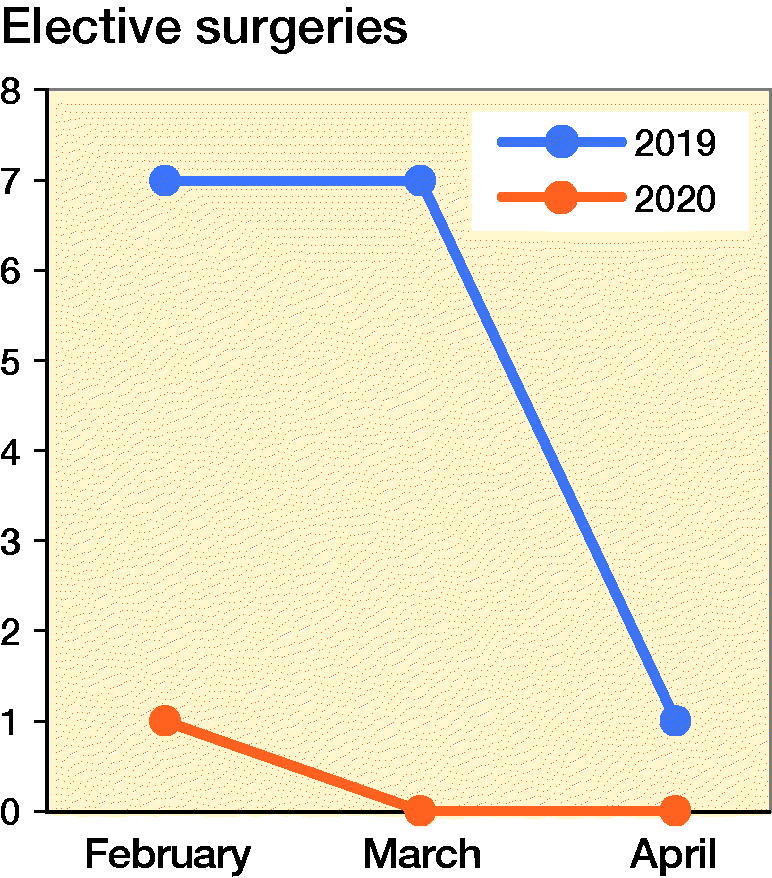
Number of elective surgeries from February to April, 2019 and 2020.

**Figure 3.**
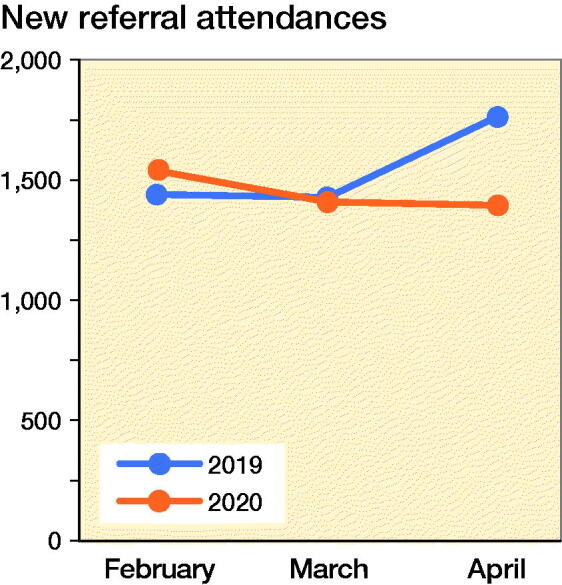
Number of new referral clinic attendances from February to April, 2019 and 2020.

There was a substantial decrease in elective surgeries by 93% and a corresponding 5% for trauma/infection surgeries (Figure 2). This is a great reduction in the exposure to elective surgery. However, the trauma and infection surgeries continued at a similar rate compared with the same period in 2019 (Figure 3). This enabled my continued training in these subspecialties. For a junior resident, this exposure is suitable. However, the same situation would not benefit a senior resident who had already completed his or her rotation in these subspecialties. The time needed to observe properly the gowning process, personnel movement before and after patient intubation, and the actual surgery itself using N95 masks, and a powered air purifying respirator (PAPR) are new challenges to the surgical team. Specialists were expected to perform surgery on positively identified or high-risk patients to minimize operative time and reduction of disease transmission. Hence in spite of the lower surgical caseload and reduced time constraints, proper patient care and appropriate risk mitigation at this juncture still supersedes my opportunity for hands-on surgical training. When an appropriate surgery for training is identified and this is triaged as a low-risk case with no history or signs of the disease, I took a meticulous approach to preoperative planning and postoperative management. These were aspects of surgery that were not emphasized as strongly in busier times.

### Specialist outpatient clinic

**Figure 4.**
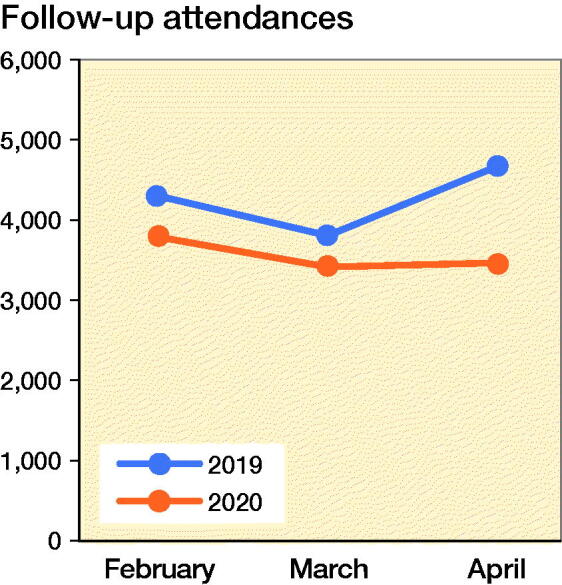
Number of follow-up clinic attendance from February to April, 2019 and 2020.

The trend in clinic attendance reflects a similar decline to that of elective surgeries. There is an overall decline in the number of new referrals and follow-up patients across the same period in 2019 and 2020. Long-term follow-up of patients at 6 months to 1 year were deferred after the diligent screening of their medical histories. There has also been a significant number of no-shows. New referrals were further categorized into trauma/infection and elective conditions. The numbers of trauma/infectious conditions requiring surgery, conversely, did not mirror the decline in clinic attendances (Figures 3 and 4) and continued to serve the needs of my residency training. This is aligned with the fact that orthopedic surgery continues to provide an essential service during a pandemic, dealing with fractures and infectious conditions.

Should this pandemic persist, it could lead to an unavoidable skew in my clinical exposure towards trauma and musculoskeletal infections and away from arthroplasty and arthroscopy conditions.

### Inpatient

When attending to a new admission, it would be necessary to exclude the possibility of a concomitant COVID-positive infection. There exists the need to consistently update oneself on the evolving screening criteria such as travel history, contact history, symptoms of acute respiratory infection, and fever. The advised level of precautionary measure needs to be observed as this prevents transmission of infection between patients and healthcare workers. The use of N95 masks and PAPR is reinforced for suspected and confirmed cases. A high index of suspicion is needed as the history of the patient might be unreliable and there are reported cases of “clean” surgical patients who were diagnosed COVID-positive after surgery was performed.

Team segregation entailed reorganizing the department staff into more teams. This change in manpower structure stretched the work capacity of junior doctors. Segregation of doctors into smaller teams to minimize physical contact mitigates risk of transmission. Advantages include facilitation of contact tracing and ability to quarantine smaller groups of healthcare providers in the event of disease transmission. Adapting to these changes, I personally made multiple check-backs on treatment instructions to ensure that patient care is not compromised.

### Didactic teaching

**Figure 5.**
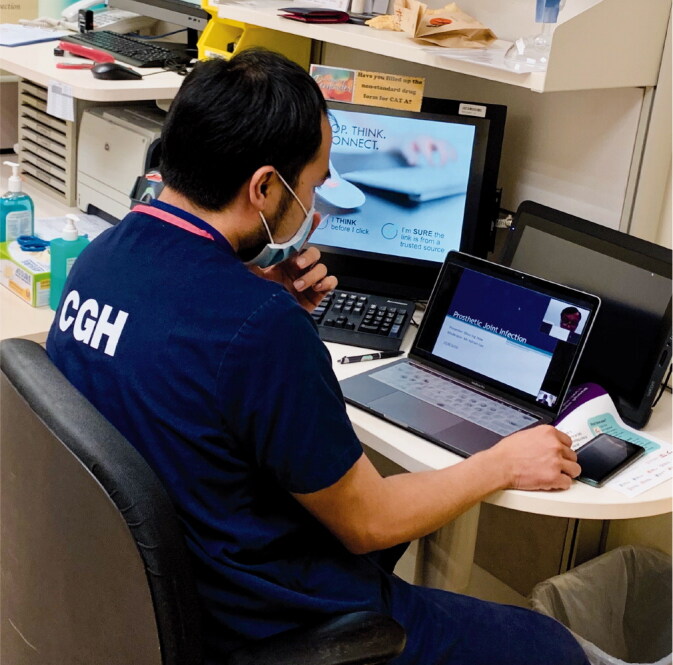
Resident conducting an interactive tutorial via teleconferencing.

**Figure 6.**
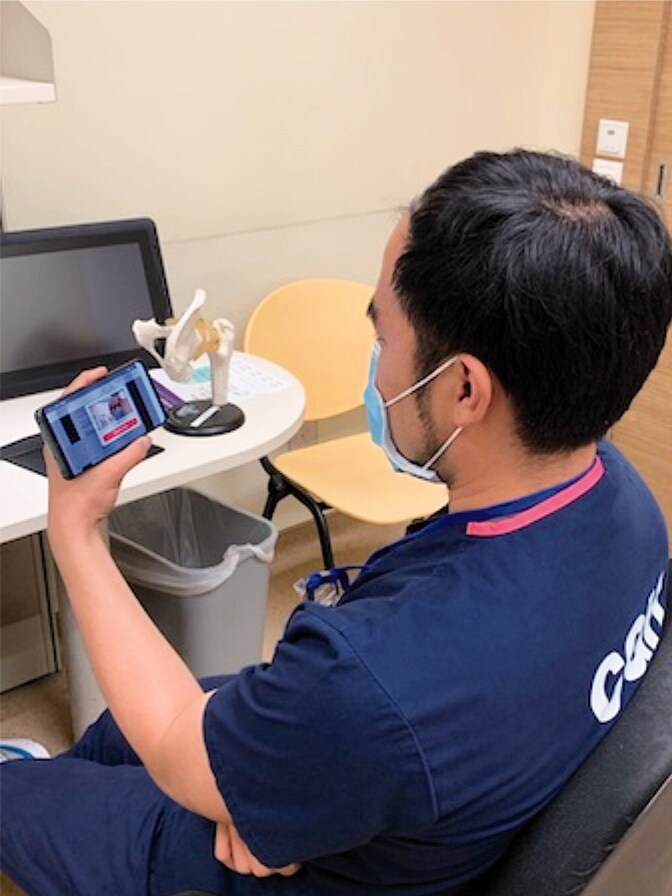
Resident conducting interactive session with supervising consultant.

Orthopedic residency requirements (ACGME 2020a) dictate 4 hours a week of didactic teaching sessions on various topics such as operative techniques, anatomy, pathology, biomechanics, and radiography. Conferences, workshops, didactic lectures, trauma rounds, and peer-reviewed learning sessions contribute to this weekly requirement. These teaching sessions involve a robust interaction between the residents and faculty. The need for social distancing has curtailed these meetings. Teleconferencing (Denstadli et al. 2012) in medicine (Lamba 2019) is one such measure that enables interactive sessions to be conducted (Figure 5). Third-party software enables physical distancing and the broadcasting of clinical cases for discussion (Figure 6). There are many teething problems in the setting up of this form of communication to meet the demands of a large department on a daily basis. One of the main concerns is ensuring security of information and abiding by the law in terms of the Personal Data Protection Act (PDPA), which came into full effect in July 2014 in Singapore. Such sessions have shown effective delivery of information and enabled robust discussions. The schedule is published in advance to enable preparation and subsequent effective discussion. However, there is a need to circumvent challenges such as speaker inaudibility caused by speaking distance from microphones, statics, and muffling by masks. Having participated in these early sessions, I feel that an interactive educational platform enhances the learning process as opposed to broadcast didactic lectures.

### Residency scholarly activities

The orthopedic resident is required to complete a series of mandatory courses locally. Training resources such as cadaveric arthroplasty (James et al. 2020) and arthroscopic surgery courses that require physical participation had to cease. Conferences worldwide have been postponed and the opportunities to make presentations are now void. Various conference organizers and scientific groups have made learning possible via alternative options such as webinars (AO Foundation 2020). Research not involving patient contact such as cadaver-based studies is an option if the resource is available. The reduction in workload potentially frees up the faculty to mentor activities such as research and fellowship applications.

### Duty hours

The manpower demand is erratic and thus carries the risk of exceeding the stipulated limit on duty hours in a working week. This situation was avoided due to close monitoring of the duty roster and strict adherence to the residency guidelines by a dedicated program director.

**Figure 7.**
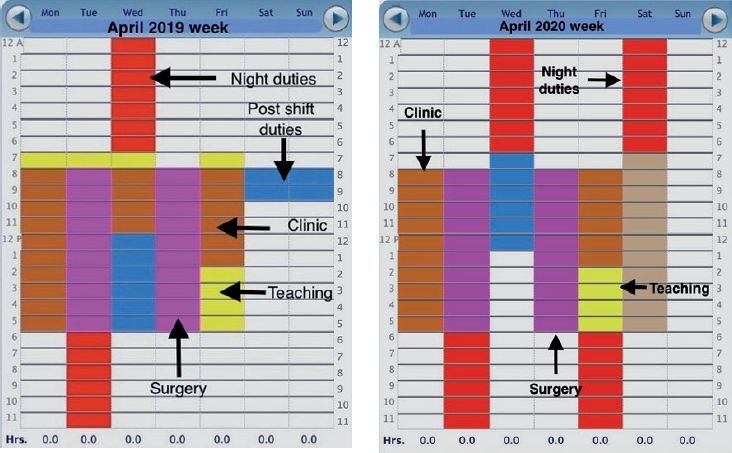
Resident’s work week schedule in April 2019 (left panel) and 2020 (right paenl).

Figure 7 illustrate an increase in overnight duties (shown in red) to compensate for colleagues deployed to the frontline. There is an overall decrease in the number of teaching sessions (in yellow). Scheduled teaching hours once a week are still maintained, albeit with online teaching resources. Allocated time for surgery (in magenta) remained unaffected. 

## The 6 competencies

### 1. Patient care

New guidelines (WHO 2020) for personal protection and surveillance of patients in both emergency and elective settings are being implemented following WHO recommendations. Platforms for telemedicine consultation for non-urgent cases have received unprecedented attention and resources to boost their capability and capacity. This form of treatment needs to be adopted promptly to provide an acceptable level of consultation without physical physician–patient contact, while bearing in mind the importance of personal data security and effective delivery of patient care.

The physical and mental health of the resident is potentially at risk (Kim et al. 2019). There is uncertainty in light of the tightening social constraints and no end in sight. The gnawing concern in relation to contracting COVID-19 in daily activities poses additional strain on pre-existing work stress. The Trauma Recovery and Counselling Services (TRACS) had raised awareness of burnout and provided counselling and stress-coping tools.

A recent survey conducted in a separate Singapore healthcare institution studied the experiences of its medical and surgical residents during the initial phase of this outbreak. Residents reported adverse effects on their medical training and career. They also reported an increase in the level of stress and burnout, citing an average of 4.7 on a scale of 0 (no stress at all) to 10 (extreme level of stress) (Wong et al. 2020). Factors such as long hours away from family and partners, freezing of non-essential annual leave entitlement, and frequent shifts at work are major contributory factors.

Rest and recreation have always been crucial in preserving the work–life balance. Team-based (Brinkley et al. 2016) physical activities amongst colleagues have been shown to benefit cohesion and organizational performance. Weekly events such as football with fellow residents have now stopped. I maintain fitness and health via individual-based exercises such as running. The study on social distancing during running (Blocken et al. 2020) appears to be common knowledge to most runners.

### 2 and 4. Knowledge, practice-based learning

Self-directed learning (Merriam 2001) is always an essential part of education. The resident needs to keep abreast of the evolving knowledge of COVID-19 disease such as its clinical course as well as management (Balla et al. 2020, Lauer et al. 2020). The intelligent sourcing and sharing of learning resources is beneficial to all involved. The same applies to orthopedic training. Resources such as the surgical skills laboratory (Sonnadara et al. 2011), cadaveric dissection, and arthroscopic simulation Gomoll et al. 2007) need to be explored. I have spent time in the surgical skills laboratory honing skills such as knot tying in shoulder arthroscopy.

### 3 and 5. Systems-based practice, interpersonal and communication skills

Residents needs to work effectively in a multidisciplinary team approach such as deployment to the intensive care unit or the emergency medicine department. The grounding in anesthesia and emergency medicine in R-1 makes them more prepared than a colleague without such prior exposure. The need to communicate effectively is tested. I take this as an opportunity to assess and improve on interpersonal and communication skills, as these are definitely applicable in future.

### 6. Professionalism

The core value of professionalism (Stern 2005) derives from the universality of disease and begins with caring and compassion. Residents make up a significant proportion of the medical community and are now called to action to support their colleagues at the frontline such as attending to intensive care patients. I need to handle the anxiety and uncertainty prevailing in the current environment and execute a treatment plan effectively, alongside compassion for the patient. Such a situation requires true professionalism and I gain insight into what it takes to do so.

## The future

Looking ahead to the near future, ramifications of the pandemic have started to impact on my career progression towards obtaining qualifications as a board-certified specialist. At the point of writing, the annually held qualification examination (Fellowship of the Royal College of Surgeons—Orthopaedic Surgery) had been announced to be postponed to a later date. A direct result is a delay in career progression with repercussions of delayed employment in my institution and associated monetary and opportunity costs. The mid-term effects of COVID-19 in the year ahead have manifested as an extension in the total duration of surgical training.

The extension of training has significant psychological bearing on my outlook and morale, and several prior personal and professional engagements have suffered from this disruption.

On an optimistic note, the training program directives, mandatory competencies, and courses have come under urgent review to lessen the impact of these disruptions. A proportion of courses have been converted to web-based sessions. Utilizing a different delivery vehicle, these sessions continue to enable residents to participate in mandatory courses. A pragmatic approach taken via a two-way communication model enables residents and faculty to identify early potential obstacles and institute appropriate changes. There is no suggestion of relaxation in these segregation measures in the near future, thus it is hopeful that these changes will continue to evolve and serve the needs of my surgical training.

## Conclusion

The COVID-19 pandemic is undoubtedly challenging in many aspects and the measures taken to facilitate training will definitely find their place in a less turbulent time in the future. There are learning points from my own experience. The ability of the program to navigate this pandemic is a credit to its robust foundation and more importantly its people. The determination of the faculty that adapted and innovated to continue the training is a reflection of their passion for education. The values of the six competencies are truly learnt and appreciated in such a trying situation. The pandemic is testing the resilience and fortitude of the orthopedic surgeon who rises to the occasion. I also see the part he can play as an orthopedic surgeon in the care of any patient, regardless of medical pathology, and hopes to look back on this pandemic with a wiser perspective on medicine. As a senior once shared with me: “First a doctor, then a surgeon.”

## Disclosure

The authors have no conflicts of interest to declare. All authors received no form of funding.

Accreditation Council for Graduate Medical Education. ACGME common program requirement (residency). 2020b. https://www.acgme.org/Portals/0/PFAssets/ProgramRequirements/CPRResidency2019.pdf (accessed April 16, 2020).
